# Bedaquiline, moxifloxacin, pretomanid, and pyrazinamide during the first 8 weeks of treatment of patients with drug-susceptible or drug-resistant pulmonary tuberculosis: a multicentre, open-label, partially randomised, phase 2b trial

**DOI:** 10.1016/S2213-2600(19)30366-2

**Published:** 2019-12

**Authors:** Conor D Tweed, Rodney Dawson, Divan A Burger, Almari Conradie, Angela M Crook, Carl M Mendel, Francesca Conradie, Andreas H Diacon, Nyanda E Ntinginya, Daniel E Everitt, Frederick Haraka, Mengchun Li, Christo H van Niekerk, Alphonse Okwera, Mohammed S Rassool, Klaus Reither, Modulakgotla A Sebe, Suzanne Staples, Ebrahim Variava, Melvin Spigelman

**Affiliations:** a MRC Clinical Trials Unit at UCL, London, UK; b University of Cape Town Lung Institute, Cape Town, South Africa; c Division of Pulmonology, Department of Medicine, University of Cape Town, Cape Town, South Africa; d Department of Statistics, University of Pretoria, Pretoria, South Africa; e TB Alliance, Pretoria, South Africa; f Global Alliance for TB Drug Development, New York, NY, USA; g Clinical HIV Research Unit, University of Witwatersrand, Johannesburg, South Africa; h TASK Applied Science, Bellville, South Africa; i Division of Physiology, Department of Medical Biochemistry, Stellenbosch University, Tygerberg, South Africa; j NIMR-Mbeya Medical Research Centre, Mbeya, Tanzania; k Ifakara Health Institute Bagamoyo Research and Training Center, Bagamoyo, Tanzania; l Uganda Case Western Reserve University Research Collaboration, Kampala, Uganda; m Clinical HIV Research Unit, Helen Joseph Hospital, Johannesburg, South Africa; n Swiss Tropical and Public Health Institute, Basel, Switzerland; o The Aurum Institute, Tembisa Hospital, Tembisa, South Africa; p THINK, Durban, South Africa; q MDR Unit, Klerksdorp Tshepong Hospital, Klerksdorp, South Africa

## Abstract

**Background:**

New anti-tuberculosis regimens that are shorter, simpler, and less toxic than those that are currently available are needed as part of the global effort to address the tuberculosis epidemic. We aimed to investigate the bactericidal activity and safety profile of combinations of bedaquiline, pretomanid, moxifloxacin, and pyrazinamide in the first 8 weeks of treatment of pulmonary tuberculosis.

**Methods:**

In this multicentre, open-label, partially randomised, phase 2b trial, we prospectively recruited patients with drug-susceptible or rifampicin-resistant pulmonary tuberculosis from seven sites in South Africa, two in Tanzania, and one in Uganda. Patients aged 18 years or older with sputum smear grade 1+ or higher were eligible for enrolment, and a molecular assay (GeneXpert or MTBDR*plus*) was used to confirm the diagnosis of tuberculosis and to distinguish between drug-susceptible and rifampicin-resistant tuberculosis. Patients who were HIV positive with a baseline CD4 cell count of less than 100 cells per uL were excluded. Patients with drug-susceptible tuberculosis were randomly assigned (1:1:1) using numbered treatment packs with sequential allocation by the pharmacist to receive 56 days of treatment with standard tuberculosis therapy (oral isoniazid, rifampicin, pyrazinamide, and ethambutol; HRZE), or pretomanid (oral 200 mg daily) and pyrazinamide (oral 1500 mg daily) with either oral bedaquiline 400 mg daily on days 1–14 then 200 mg three times per week (B_load_PaZ) or oral bedaquiline 200 mg daily (B_200_PaZ). Patients with rifampicin-resistant tuberculosis received 56 days of the B_200_PaZ regimen plus moxifloxacin 400 mg daily (BPaMZ). All treatment groups were open label, and randomisation was not stratified. Patients, trial investigators and staff, pharmacists or dispensers, laboratory staff (with the exception of the mycobacteriology laboratory staff), sponsor staff, and applicable contract research organisations were not masked. The primary efficacy outcome was daily percentage change in time to sputum culture positivity (TTP) in liquid medium over days 0–56 in the drug-susceptible tuberculosis population, based on non-linear mixed-effects regression modelling of log_10_ (TTP) over time. The efficacy analysis population contained patients who received at least one dose of medication and who had efficacy data available and had no major protocol violations. The safety population contained patients who received at least one dose of medication. This study is registered with ClinicalTrials.gov, NCT02193776, and all patients have completed follow-up.

**Findings:**

Between Oct 24, 2014, and Dec 15, 2015, we enrolled 180 patients with drug-susceptible tuberculosis (59 were randomly assigned to B_load_PaZ, 60 to B_200_PaZ, and 61 to HRZE) and 60 patients with rifampicin-resistant tuberculosis. 57 patients in the B_load_PaZ group, 56 in the B_200_PaZ group, and 59 in the HRZE group were included in the primary analysis. B_200_PaZ produced the highest daily percentage change in TTP (5·17% [95% Bayesian credibility interval 4·61–5·77]), followed by B_load_PaZ (4·87% [4·31–5·47]) and HRZE group (4·04% [3·67–4·42]). The bactericidal activity in B_200_PaZ and B_load_PaZ groups versus that in the HRZE group was significantly different. Higher proportions of patients in the B_load_PaZ (six [10%] of 59) and B_200_PaZ (five [8%] of 60) groups discontinued the study drug than in the HRZE group (two [3%] of 61) because of adverse events. Liver enzyme elevations were the most common grade 3 or 4 adverse events and resulted in the withdrawal of ten patients (five [8%] in the B_load_PaZ group, three [5%] in the B_200_PaZ group, and two [3%] in the HRZE group). Serious treatment-related adverse events affected two (3%) patients in the B_load_PaZ group and one (2%) patient in the HRZE group. Seven (4%) patients with drug-susceptible tuberculosis died and four (7%) patients with rifampicin-resistant tuberculosis died. None of the deaths were considered to be related to treatment.

**Interpretation:**

B_200_PaZ is a promising regimen to treat patients with drug-susceptible tuberculosis. The bactericidal activity of both these regimens suggests that they have the potential to shorten treatment, and the simplified dosing schedule of B_200_PaZ could improve treatment adherence in the field. However, these findings must be investigated further in a phase 3 trial assessing treatment outcomes.

**Funding:**

TB Alliance, UK Department for International Development, Bill & Melinda Gates Foundation, US Agency for International Development, Directorate General for International Cooperation of the Netherlands, Irish Aid, Australia Department of Foreign Affairs and Trade, and the Federal Ministry for Education and Research of Germany.

## Introduction

Tuberculosis is the leading cause of death globally from an infectious disease.^1^ The treatment for drug-susceptible tuberculosis has not changed for two decades and continues to be based on isoniazid and rifampicin given for at least 6 months, supplemented by pyrazinamide and ethambutol given in the first 2 months (HRZE).^2^ The length of therapy and associated logistical challenges, along with recognised treatment-associated toxicity, result in favourable outcomes in approximately 80% of people with drug-susceptible tuberculosis in real-world settings.^1^

The HIV epidemic and the emergence of multidrug-resistant tuberculosis continue to act as major drivers of the tuberculosis epidemic.^3^ Patients with tuberculosis and HIV coinfection are at greater risk of treatment failure^4^, ^5^ and higher treatment-related toxicity from tuberculosis medication than those with tuberculosis only, with the potential for overlapping toxicity from antiretroviral therapy (ART).^6^, ^7^ Multidrug-resistant tuberculosis is a disease with strains resistant to both rifampicin and isoniazid, and a 2018 communication^8^ from WHO recommends treating both multidrug-resistant and rifampicin-resistant tuberculosis with either a 20–24-month all-oral regimen or a shorter 9–11-month regimen for patients with confirmed fluoroquinolone and aminoglycoside sensitivity. The short regimen still requires a minimum 4 months of treatment in an intensive phase using an aminoglycoside (amikacin) with the consequent risk of ototoxicity, renal toxicity, and logistical challenges of parenteral drug administration. The 20–24-month oral regimen consists of five drugs: levofloxacin or moxifloxacin, bedaquiline, linezolid, clofazimine, and cycloserine or terizidone. Further drugs can be added from an approved list to complete the regimen if any of these drugs are contraindicated.

**Research in context**

**Evidence before this study**

We searched PubMed for clinical trials published between Jan 1, 1970, and Dec 31, 2015, studying tuberculosis drug regimens reporting sputum culture results during the first 2 months of therapy. PubMed search terms used included “pulmonary tuberculosis” AND “antituberculosis” AND “two-month sputum culture-negativity” OR “sputum culture-positivity” AND “serial mycobacterial culture” AND “fluoroquinolones” AND “bedaquiline” AND “relapse rates”. Fluoroquinolones have been studied in both phase 2 and phase 3 studies of antituberculosis therapy previously as substitutions for one of the standard first-line therapy drugs. Phase 2b studies have been done of moxifloxacin, pretomanid, and pyrazinamide in different combinations, and of bedaquiline as adjuvant therapy to standard five-drug second-line tuberculous treatment. A regimen comprising bedaquiline, pretomanid, and clofazimine was previously studied in a phase 2a trial.

**Added value of this study**

This trial was the first phase 2b study investigating the efficacy and safety of the combination therapy of bedaquiline, pretomanid, and pyrazinamide, with or without moxifloxacin in patients with drug-susceptible and multidrug-resistant tuberculosis. The combination therapy of bedaquiline, pretomanid, and pyrazinamide has not been investigated elsewhere except in a phase 2a study sponsored by TB Alliance. This phase 2b study was also the first to investigate two different dosing schedules of bedaquiline.

**Implications of all the available evidence**

In patients with tuberculosis, the combination therapy of bedaquiline, pretomanid, and pyrazinamide, with or without moxifloxacin, had superior bactericidal activity during the first 8 weeks of treatment compared with standard treatment of isoniazid, rifampicin, pyrazinamide, and ethambutol. Simplified, once-daily dosing for bedaquiline showed bactericidal activity similar to the established dosing schedule. Bedaquiline, pretomanid, and pyrazinamide plus moxifloxacin and bedaquiline, pretomanid, and pyrazinamide hold promise for outcomes superior to the standard therapy for multidrug-resistant tuberculosis and drug-susceptible tuberculosis, respectively. These regimens can now be taken forward to phase 3 studies (to be assessed for improvement in tuberculosis treatment outcomes as measured by shortening of tuberculosis treatment duration and simplification of regimens for drug-susceptible and multidrug-resistant tuberculosis).

Much shorter, efficacious, and better-tolerated oral regimens than are currently available are required to tackle the ongoing epidemic in both drug-susceptible tuberculosis and multidrug-resistant and rifampicin-resistant tuberculosis. Fluoroquinolones have been studied previously for the first 2 months of antituberculosis treatment as substitutions for one of the standard first-line therapy drugs.^9^, ^10^, ^11^, ^12^ Combination therapy of moxifloxacin with pretomanid and pyrazinamide was studied during an 8-week phase 2b study.^13^ Bedaquiline has been studied as an adjuvant therapy to the standard five-drug second-line antituberculosis regimen in a 2-month phase 2b trial.^14^ A novel regimen of bedaquiline, pretomanid, pyrazinamide, and clofazimine was previously studied in a phase 2a trial.^15^

We aimed to determine the safety and efficacy of 8-week treatment with the combination therapy of bedaquiline, pretomanid, and pyrazinamide (BPaZ) compared with standard tuberculosis therapy in patients with newly diagnosed drug-susceptible tuberculosis using two different dosing strategies for bedaquiline. We also assessed the response in patients with rifampicin-resistant tuberculosis over 8 weeks with the addition of moxifloxacin to a BPaZ regimen (BPaMZ), to compensate for possible pyrazinamide resistance in these patients.

## Methods

### Study design

NC-005 was a multicentre, open-label, partially randomised, phase 2b trial done in seven sites in South Africa, two sites in Tanzania, and one site in Uganda. All sites adopted outpatient management, with the exception of the site in Uganda, where patients with rifampicin-resistant tuberculosis were admitted for the first week (approximately) of treatment, in keeping with existing local practice for patients with rifampicin-resistant tuberculosis.

The study was approved by the applicable national and local ethics committees at the different sites. A list of the ethics committees can be found in the appendix (pp 2–3). The use of pretomanid in drug-susceptible tuberculosis was ethically justified based on previous experience with the drug in phase 2 and 3 trials, and in light of the acknowledged need for shorter treatment regimens in drug-susceptible disease that are not impaired in the presence of isoniazid monoresistance. The protocol is available online.

### Patients

Patients aged 18 years or older with positive direct sputum microscopy for acid-fast bacilli, grade 1+ or higher on the WHO/International Union Against Tuberculosis and Lung Disease scale,^16^ were eligible for inclusion in the study. The full list of inclusion and exclusion criteria can be found in the appendix (pp 4–6). Patients included in the drug-susceptible tuberculosis cohort had strains susceptible to isoniazid and rifampicin and those included in the rifampicin-resistant tuberculosis cohort had strains resistant to rifampicin but sensitive to fluoroquinolones.

A molecular assay was used for identification of *Mycobacterium tuberculosis* and drug susceptibility (GeneXpert [Cepheid, Sunnyvale, CA, USA] or MTBDR*plus* [Hain Lifescience, Nehren, Germany]) to confirm the diagnosis of tuberculosis, and to distinguish between drug-susceptible tuberculosis and rifampicin-resistant tuberculosis; cases of rifampicin-resistant tuberculosis were further tested for fluoroquinolone resistance using a molecular platform (MTBDR*sl* [Hain Lifescience]). MTBDR*plus* was used to detect isoniazid and rifampicin resistance, where available.

Patients with HIV were eligible if their CD4 cell count was higher than 100 cells per μL at baseline. All patients had their baseline CD4 cell count checked because it was an exclusion criterion; however, viral load was not routinely checked. The site doctor was responsible for ART of HIV-positive patients in the study, and ART use was encouraged for all HIV-positive patients. Permitted ART regimens in the study are presented in the exclusion criteria in the appendix (p 5). However, if the patient was not already taking a permitted ART regimen, a 2-week interval was required after starting trial medication and initiating ART to reduce the risk of immune reconstitution inflammatory syndrome. The protocol did not require that HIV-positive patients were initiated on ART, and the final judgment was left to the site doctor managing the patient.

Patients were excluded from the study if there was evidence of clinically significant extrathoracic tuberculosis, which would have been clinically evaluated by the site doctor. Other exclusion criteria included risks for cardiac arrhythmia (eg, QTc interval >450 ms), alanine aminotransferase (ALT) or aspartate aminotransferase (AST) concentrations of 3 × upper limit of normal (ULN) or more, alkaline phosphatase concentration of more than 8 × ULN, and total bilirubin concentration of 2 × ULN or more at baseline. All patients provided written or oral, witnessed, informed consent before any screening procedures were carried out. A patient information sheet was provided and translated into appropriate languages.

### Randomisation and masking

Patients with drug-susceptible tuberculosis were randomly assigned (1:1:1) to receive one of three treatment regimens: two experimental BPaZ regimens or standard tuberculosis therapy as a control. The randomisation list was generated by people not directly involved with the trial. All treatment groups were open label. Patients, trial investigators and staff, pharmacists or dispensers, laboratory staff (with the exception of the mycobacteriology laboratory staff), sponsor staff, and applicable contract research organisations were not masked.

A treatment pack was available for each patient and was identified by a treatment number. To ensure that there was no bias in treatment assignment, the randomisation list was retained by the pharmacist or registered dispenser. The person responsible for overseeing the random assignment for the drug-susceptible tuberculosis cohort and allocation for the rifampicin-resistant cohort was not directly involved in patient care.

At the time of assignment, the trial centre requested the pharmacist or registered dispenser to assign a study drug treatment pack to the patient depending on their drug susceptibility or multidrug-resistant tuberculosis status. The pharmacist or registered dispenser accordingly assigned the patient the next available applicable treatment pack, sequentially starting from the lowest unused treatment number. A cohort of patients with rifampicin-resistant tuberculosis was allocated to receive BPaMZ.

### Procedures

Patients with drug-susceptible tuberculosis in the B_load_PaZ and B_200_PaZ groups received pretomanid oral 200 mg and pyrazinamide oral 1500 mg once daily on days 1–56 with bedaquiline. Patients in the B_load_PaZ group received oral bedaquiline 400 mg once daily for the first 14 days, and oral 200 mg three times per week for the remaining days; those in the B_200_PaZ group received oral bedaquiline 200 mg once daily throughout the 8 weeks of therapy. Patients in the control group were prescribed fixed-dose combination pills containing isoniazid, rifampicin, pyrazinamide, and ethambutol (HRZE) on the basis of their weight at baseline (HRZE tablets [MacLeods Pharmaceuticals, Mumbai, India]).

Patients with rifampicin-resistant tuberculosis were allocated to receive treatment with oral bedaquiline 200 mg, oral pretomanid 200 mg, oral pyrazinamide 1500 mg, and oral moxifloxacin 400 mg (BPaMZ) daily for 8 weeks. Adherence to outpatient treatment was based on patient reporting; site staff recorded adherence if the patient was treated in hospital. For details on drug dosing, see the appendix (p 7).

Overnight sputum samples were collected from patients before randomisation (day −1 and −2) and days 1, 3, 7, 14, 21, 28, 35, 42, 49 and 56 after randomisation. Colony-forming unit (CFU) counts on solid media and time to sputum culture positivity (TTP) in Mycobacterial Growth Indicator Tube (MGIT; BD, Franklin Lakes, NJ, USA) on liquid media (ie, the time it takes for a sputum culture to grow positive for tuberculosis [expressed in hours]) were measured on all sputum samples. TTP was specified as the primary readout in all analyses.

Patients enrolled in the study attended scheduled visits at each site between day 1 and day 140 (see appendix p 8 for full visit schedule). Electrocardiograms (ECGs) were carried out routinely during screening, and on days 1, 15, 29, 43, 57, and 70. All patients were referred to the national tuberculosis programme of the country where they were being treated for further treatment after finishing their study-allocated treatment, but the survival status of patients was checked by phone calls at months 8, 14, 20, and 26. Full blood count, clinical chemistry, and liver biochemical tests were done at screening and days 1, 8, 15, 22, 29, 36, 43, 50, 57 and 70.

Adverse events were classified according to the Division of Microbiology and Infectious Disease Adult Toxicity Table^17^ as a numerical score for assessing the severity of the event. The site doctor was to actively enquire about any adverse medical events that the patient had experienced at every visit (including assessing whether previously recorded events had worsened or improved) and to record event severity, date of onset and stop date (if applicable), action taken with study medication in response to the event, and relationship to study medication.

The attribution of relatedness was ultimately left to the clinical judgment of the site doctor with guidance provided to assess the event as one of “not related”, “unlikely related”, “possibly related”, “probably related”, or “definitely related”, and in the final dataset any event assessed as possibly, probably, or definitely related was considered “related”.

An independent data safety management committee met twice during the study to review unblinded aggregate data and provided feedback to the sponsor. On both of these occasions, the recommendation was to continue without any change to the trial design.

### Outcomes

The primary efficacy outcome was the daily percentage change in TTP over days 0–56 in the drug-susceptible tuberculosis population, based on non-linear mixed-effects regression modelling of log_10_(TTP) over time. For all efficacy outcomes, the primary analysis was based on cultures of overnight sputum samples.

A secondary efficacy endpoint was the time to sputum culture conversion in solid and liquid media in patients with drug-susceptible tuberculosis and in those with rifampicin-resistant tuberculosis. Other secondary efficacy endpoints included the daily rate of decline in log_10_ (CFU) count over days 0–56 and a subgroup analysis comparing the bactericidal activity among pyrazinamide-resistant versus pyrazinamide-susceptible infections in patients with rifampicin-resistant tuberculosis, and among HIV-positive and HIV-negative patients (see appendix p 10 for the full list of secondary endpoints). The safety analysis included the incidence of treatment emergent adverse events and abnormal laboratory tests for each treatment group. In addition, ECG findings, including QT and QTc intervals, for patients in each treatment group were also analysed.

### Statistical analysis

For this study, the sample size calculation was based on an earlier study^1^ that reported mean rate constants from a non-linear mixed-effects regression model for the rate of decline in CFU count over 56 days of treatment. On the basis of a sample of 50 patients per treatment group (given a one-sided significance level of 2·5%), the statistical power to yield a significant difference between groups was 86% for second-phase (or terminal-phase) response.^9^, ^13^ All statistical tests were two-sided with a significance level of 5%. We did no multiplicity adjustments for this study. Descriptive statistics included the number (%) of patients, mean (SD), and minimum, median (IQR), and maximum where applicable.

The safety analysis population contained patients who received at least one dose of study drug. The efficacy analysis population contained patients included in the safety analysis population for whom efficacy data were available and who had no major protocol violations that could affect the integrity of the data, such as those relating to eligibility criteria or ethics approvals. All analyses are presented without adjustment for baseline covariates as randomisation was not stratified.

We estimated the bactericidal activity parameters by the fit of a Bayesian non-linear mixed-effects regression model to log_10_(TTP)^18^ and log_10_(CFU) count^19^, ^20^ of all patients with drug-susceptible tuberculosis jointly. The bactericidal activity was characterised by the daily percentage change in time to positive signal, and the daily change in log(CFU) of overnight sputum samples. The posterior estimates and corresponding 95% Bayesian credibility intervals (BCIs) for the mean bactericidal activity characteristics are presented. We did pairwise comparisons of the B_200_PaZ and B_load_PaZ groups against the HRZE group to investigate bactericidal activity of the experimental treatment groups.

We did a Kaplan-Meier analysis to test the difference in the cumulative proportion of patients with drug-susceptible tuberculosis who were culture negative, in solid and liquid media, at 8 weeks in the experimental treatment groups versus the HRZE group. We compared the median time to culture conversion in the B_200_PaZ and B_load_PaZ groups versus the HRZE group using the log-rank test.

We calculated the corresponding hazard ratios for time to negative culture in liquid and solid culture using Cox proportional hazards regression modelling without the adjustment of covariates. Model assumptions were tested to ensure that there were no violations, and censoring occurred at 8 weeks or if the patient was withdrawn from the study. The proportion of patients within each category of adverse events was compared between treatments using the Freeman-Halton test in a post-hoc analysis. All analyses were done with SAS (version 9.4), OpenBUGS (version 3.1.3), and R (version 3.0.2). This study is registered with ClinicalTrials.gov, NCT02193776.

### Role of funding source

The funders of the study were involved in study design, data collection, data analysis, data interpretation, and writing of this report. Monitoring visits were carried out by the monitoring clinical research organisation appointed by TB Alliance. Monitoring took place according to the monitoring plan (see protocol). A risk-based approach was followed with both on-site and remote monitoring at the intervals specified in the monitoring plan. Intervals depended on the stage of the study at the sites to ensure accurate data collection relating to both efficacy and safety findings. The corresponding author had full access to all the data in the study and had final responsibility for the decision to submit for publication.

## Results

**Figure 1:**
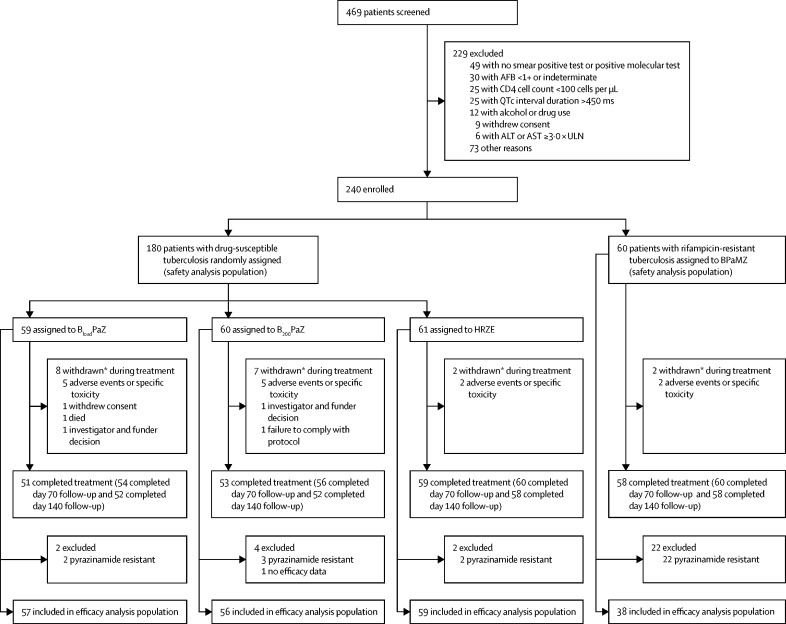
Trial profile Full listings of the reasons for screening failure are available in the appendix (p 7). AFB=acid-fast bacilli. ALT=alanine aminotransferase. AST=aspartate aminotransferase. B_load_PaZ=bedaquiline (loading dose), pretomanid, and pyrazinamide. B_200_PaZ=bedaquiline (daily dose), pretomanid, and pyrazinamide. BPaMZ=bedaquiline (daily dose), pretomanid, and pyrazinamide plus moxifloxacin. HRZE=isoniazid, rifampicin, pyrazinamide, and ethambutol. *Primary reason for withdrawal during treatment.

Between Oct 24, 2014, and Dec 15, 2015, 180 patients drug-susceptible tuberculosis were randomly assigned to the B_load_PaZ (n=59), B_200_PaZ (n=60), or HRZE (n=61) groups (figure 1). 60 patients with rifampicin-resistant tuberculosis were assigned to the BPaMZ group. Concurrent isoniazid resistance was detected in 53 (88%) of 60 rifampicin-resistant tuberculosis isolates.

182 (76%) of 240 participants were men, 197 (82%) were of black African ethnicity, and 53 (22%) patients were HIV positive (table 1). All patients had an overall adherence to study drug regimen of at least 80% of prescribed doses (table 1).

**Table 1:** Demographic and other baseline characteristics of the safety analysis population

			**Patients with drug-susceptible tuberculosis**			**Patients with rifampicin-resistant tuberculosis (BPaMZ group [n=60])**
			B_load_PaZ group (n=59)	B_200_PaZ group (n=60)	HRZE group (n=61)	
Age*, years			35·1 (13·0)	33·9 (10·5)	33·3 (8·6)	34·0 (12·7)
Sex						
	Female		14 (24%)	12 (20%)	15 (25%)	17 (28%)
	Male		45 (76%)	48 (80%)	46 (75%)	43 (72%)
Race						
	Black or African American		46 (78%)	49 (82%)	49 (80%)	53 (88%)
	Native Hawaiian or other Pacific Islander		0	0	0	1 (2%)
	White		0	0	0	1 (2%)
	Mixed race		13 (22%)	11 (18%)	12 (18%)	5 (8%)
Site						
	TASK, Cape Town		11 (19%)	12 (20%)	13 (21%)	10 (17%)
	UCTLI, Cape Town		13 (22%)	13 (22%)	13 (21%)	0
	CHIVRU, Helen Joseph		5 (9%)	5 (8%)	5 (8%)	0
	Aurum Institute, Tembisa		5 (9%)	5 (8%)	4 (7%)	0
	Tshepong, Klerksdorp		0	0	0	25 (42%)
	Ifakara, Bagamoyo		10 (17%)	10 (17%)	11 (18%)	0
	Mbeya Research Centre, Mbeya		11 (19%)	11 (18%)	10 (16%)	0
	UWCHIVRU, Johannesburg		0	0	0	2 (3%)
	THINK, Durban		4 (7%)	4 (7%)	5 (8%)	0
	Case Western, Kampala		0	0	0	23 (38%)
Weight, kg			56·1 (10·8)	54·4 (9·1)	52·7 (8·8)	50·8 (8·4)
HIV status						
	Positive		8 (14%)	10 (17%)	10 (16%)	25 (42%)
		CD4 cell count	456·5 (135·1)	264·7 (89·6)	620·6 (307·4)	400·3 (220·5)
	Negative		51 (86%)	50 (83%)	51 (84%)	35 (58%)
Antiretroviral therapy at randomisation						
	Yes		0	1 (10%)	0	16 (64%)
	No		8 (100%)	9 (90%)	10 (100%)	9 (36%)
Pyrazinamide susceptibility						
	Susceptible		57 (97%)	57 (95%)	59 (97%)	38 (63%)
	Resistant		2 (3%)	3 (5%)	2 (3%)	22 (37%)
Ethambutol susceptibility						
	Susceptible		54 (96%)	51 (93%)	57 (97%)	39 (78%)
	Resistant		2 (4%)	4 (7%)	2 (3%)	11 (22%)
	Not done		0	0	0	1 (2%)
Compliance to study drug						
	<80%		0	0	0	0
	≥80%		59 (100%)	60 (100%)	61 (100%)	60 (100%)

Data are n (%) or mean (SD). B_load_PaZ=bedaquiline (loading dose), pretomanid, and pyrazinamide. B_200_PaZ=bedaquiline (daily dose), pretomanid, and pyrazinamide. BPaMZ=bedaquiline (daily dose), pretomanid, and pyrazinamide plus moxifloxacin. CHIVRU=Clinical HIV Research Unit. HRZE=isoniazid, rifampicin, pyrazinamide, and ethambutol. UCTLI=University of Cape Town Lung Institute. UWCHIVRU=University of Witwatersrand Clinical HIV Research Unit.

* Calculated relative to informed consent.

57 patients in the B_load_PaZ group, 56 in the B_200_PaZ group, and 59 in the HRZE group were included in the primary analysis. In the efficacy analysis, patients in the B_200_PaZ group showed the highest daily percentage change in TTP (5·17% [95% BCI 4·61–5·77]), followed by those in the B_load_PaZ group (4·87% [4·31–5·47]) and those in the HRZE group (4·04% [3·67–4·42]; table 2). The differences in bactericidal activity of the B_200_PaZ and B_load_PaZ groups versus the HRZE group were significant. Descriptive statistics of log(TTP) are presented in the appendix (pp 11–13).

**Table 2:** Bactericidal activity among patients with drug-susceptible tuberculosis over days 0–56 in the efficacy analysis population

	**B**_load_**PaZ group (n=57)**	**B**_200_**PaZ group (n=56)**	**HRZE group (n=59)**
Daily percentage change in time to positive signal	4·87% (4·31–5·47)	5·17% (4·61–5·77)	4·04% (3·67–4·42)
Daily change in log(CFU) of overnight sputum samples	0·12 (0·11–0·14)	0·11 (0·10–0·12)	0·12 (0·11–0·13)

Data are posterior mean estimate (95% Bayesian credibility interval). The differences between the pairs of treatments B_load_PaZ versus HRZE and B_200_PaZ versus HRZE were significant. B_load_PaZ=bedaquiline (loading dose), pretomanid, and pyrazinamide. B_200_PaZ=bedaquiline (daily dose), pretomanid, and pyrazinamide. CFU=colony-forming unit. HRZE=isoniazid, rifampicin, pyrazinamide, and ethambutol.

**Figure 2:**
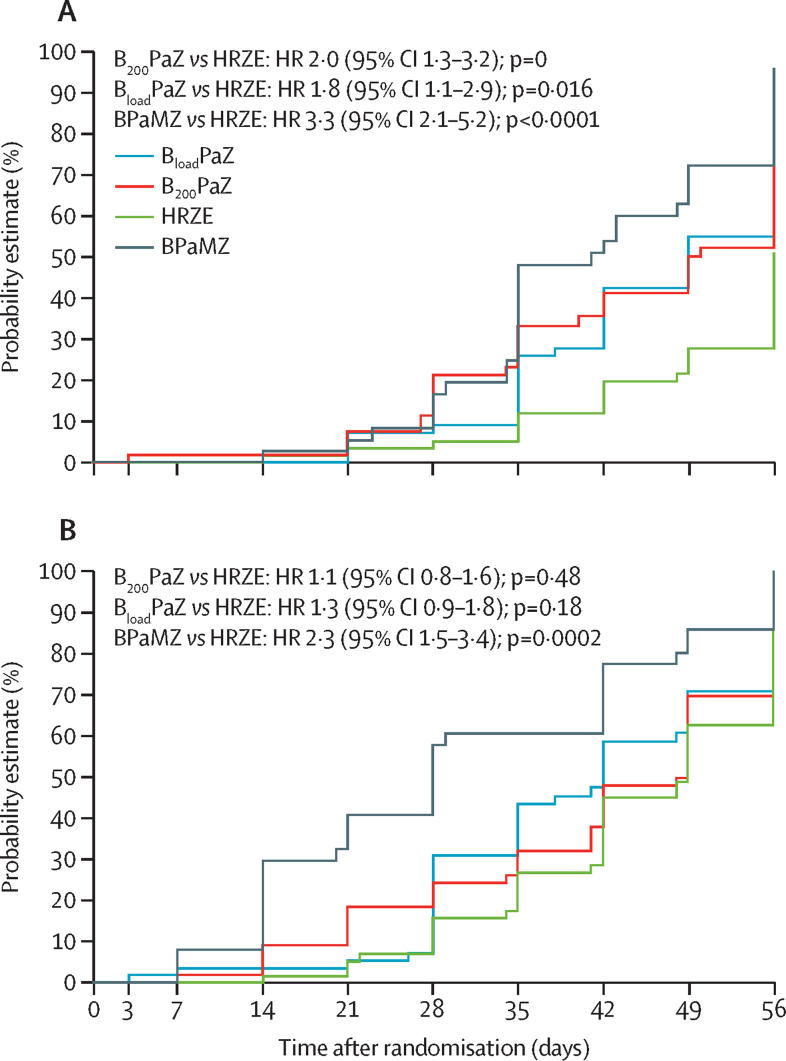
Kaplan-Meier curves of time to liquid (A) and solid (B) media sputum culture conversion the efficacy analysis population Patients censored at last available valid sample day if no sample was collected at day 56. (A) The difference between B_200_PaZ versus HRZE with respect to the cumulative probability of liquid media sputum culture conversion is significant. No other differences between treatment groups were significant. (B) No differences between treatment groups were significant with respect to the cumulative probability of solid media sputum culture conversion. p values calculated from a log-rank test for comparison of median time to liquid media sputum culture conversion. B_load_PaZ=bedaquiline (loading dose), pretomanid, and pyrazinamide. B_200_PaZ=bedaquiline (daily dose), pretomanid, and pyrazinamide. BPaMZ=bedaquiline (daily dose), pretomanid, and pyrazinamide plus moxifloxacin. HR=hazard ratio. HRZE=isoniazid, rifampicin, pyrazinamide, and ethambutol.

In the prespecified secondary analysis, among the drug-susceptible tuberculosis treatment groups, B_200_PaZ showed the highest cumulative percentage of culture negativity in liquid culture, followed by B_load_PaZ and HRZE (table 3; figure 2). In liquid culture, the corresponding HR of time to culture negative status for B_load_PaZ versus HRZE and B_200_PaZ versus HRZE was significantly higher than 1 in liquid culture (table 4).

**Table 3:** Cumulative percentage in patients with drug-susceptible tuberculosis with culture negative overnight sputum samples and the median time to sputum culture conversion in the efficacy analysis population

		**B**_load_**PaZ group (n=57)**	**B**_200_**PaZ group (n=56)**	**HRZE group (n=59)**
Percentage culture negative at day 56 of treatment (95% CI)				
	Liquid culture	67·4% (53·8–80·9)	76·1% (64·0–88·3)*	51·0% (36·6–65·4)
	Solid culture	88·9% (79·8–97·9)	84·0% (73·9–94·1)	85·5% (75·7–95·3)
Liquid culture median (IQR) time to culture negative, days		49*(35 to not obtained)	49* (35–56)	56 (49 to not obtained)

B_load_PaZ=bedaquiline (loading dose), pretomanid, and pyrazinamide. B_200_PaZ=bedaquiline (daily dose), pretomanid, and pyrazinamide. HRZE=isoniazid, rifampicin, pyrazinamide, and ethambutol.

* Significantly different to the HRZE control; the median times were compared with the HRZE control using the log-rank test.

**Table 4:** Hazard ratios for culture negative status among patients with drug-susceptible tuberculosis according to treatment group versus control group in the efficacy analysis population

	**B**_load_**PaZ group (n=57)**	**B**_200_**PaZ group (n=56)**	**HRZE group (n=59)**
Liquid culture	34 (60%); 1·8 (1·1–2·9)	37 (65%); 2·0 (1·3–3·2)	25 (42%); 1 (ref)
Solid culture	46 (81%); 1·3 (0·9–1·8)	43 (75%); 1·1 (0·8–1·6)	45 (76%); 1 (ref)

Data are n (%); hazard ratio (95% CI). The hazard ratio of time to culture negative status for B_load_PaZ versus HRZE, and B_200_PaZ versus HRZE was significantly higher than 1 in liquid culture. B_load_PaZ=bedaquiline (loading dose), pretomanid, and pyrazinamide. B_200_PaZ=bedaquiline (daily dose), pretomanid, and pyrazinamide. HRZE=isoniazid, rifampicin, pyrazinamide, and ethambutol.

In the prespecified secondary subgroup analysis in the BPaMZ group, the pyrazinamide-susceptible rifampicin-resistant tuberculosis group showed the highest cumulative percentage of culture negativity in liquid culture medium, followed by the pyrazinamide-resistant rifampicin-resistant tuberculosis group (table 5).

**Table 5:** Cumulative percentage in patients in the BPaMZ group with rifampicin-resistant tuberculosis and culture negative overnight sputum samples, and the median time to sputum culture conversion

	**Pyrazinamide-susceptible rifampicin-resistant tuberculosis (n=38)**	**Pyrazinamide-resistant**	**rifampicin-resistant tuberculosis (n=22)**
Percentage culture negative at day 56 of treatment (95% CI)			
	Liquid culture	96·0% (88·5–100·0)	79·8% (62·4–97·2)
	Solid culture	100·0% (100·0–100·0)	95·0% (85·3–100·0)
Liquid culture median (IQR) time to culture negative, days		41 (35–56)	49 (34–56)

BPaMZ=bedaquiline (daily dose), pretomanid, and pyrazinamide plus moxifloxacin.

Bactericidal activity was similar in both HIV-positive and HIV-negative patients receiving BPaMZ (appendix p 14). In our post-hoc analysis, we found no significant differences between the drug-susceptible tuberculosis treatment groups in the incidence of adverse events. The proportion of patients with drug-susceptible tuberculosis with peak ALT or AST of at least 5 × ULN was higher for the B_load_PaZ (six [10%] of 59 patients) and B_200_PaZ (four [7%] of 60 patients) groups than for patients receiving HRZE (three [5%] of 61 patients; table 6). No QT or QTc intervals of 500 ms or more were reported during treatment in the drug-susceptible tuberculosis groups. 13 patients with drug-susceptible tuberculosis were prematurely withdrawn from study drug administration due to adverse events: six (10%) of 59 patients receiving B_load_PaZ, five (8%) of 60 patients receiving B_200_PaZ, and two (3%) of 61 patients receiving HRZE. Increases in ALT or AST resulted in treatment discontinuation for ten patients (five [8%] patients in the B_load_PaZ group, three [5%] patients in the B_200_PaZ group, and two [3%] patients in the HRZE group).

**Table 6:** Adverse events in the safety analysis population

		**Patients with drug-susceptible tuberculosis**				**Patients with rifampicin-resistant tuberculosis (BPaMZ group [n=60])**
		B_load_PaZ group (n=59)	B_200_PaZ group (n=60)	HRZE group (n=61)	p value	
≥1 grade 3 treatment-emergent adverse event		19 (32%)	17 (28%)	14 (23%)	0·53	13 (22%)
≥1 grade 4 treatment-emergent adverse event		8 (14%)	7 (12%)	2 (3%)	0·11	1 (2%)
≥1 serious treatment-emergent adverse event		4 (7%)	3 (5%)	4 (7%)	0·93	4 (7%)
≥1 serious liver-related treatment-emergent adverse event		2 (3%)	0	2 (3%)	0·47	2 (3%)
≥1 treatment-emergent adverse event leading to treatment discontinuation		6 (10%)	5 (8%)	2 (3%)	0·28	2 (3%)
Deaths during treatment/total deaths (%)		1/2 (50%)	1/3 (33%)	1/2 (50%)	1·0*; 0·90†	0/4 (0%)
Liver toxicity						
	ALT or AST ≥5 × ULN	6 (10%)	4 (7%)	3 (5%)	0·48	3 (5%)
	ALT or AST ≥10 × ULN	3 (5%)	3 (5%)	1 (2%)	0·57	1 (2%)
ECG findings						
	≥60 ms increase in QTc interval from baseline	0	3 (5%)	1 (2%)	0·07	0

Data are n (%) unless otherwise stated. All patients who received at least dose of trial medication included in the analysis. ALT=alanine aminotransferase. AST=aspartate aminotransferase. B_load_PaZ=bedaquiline (loading dose), pretomanid, and pyrazinamide. B_200_PaZ=bedaquiline (daily dose), pretomanid, and pyrazinamide. BPaMZ=bedaquiline (daily dose), pretomanid, and pyrazinamide plus moxifloxacin. ECG=electrocardiogram. HRZE=isoniazid, rifampicin, pyrazinamide, and ethambutol. ULN=upper limit of normal.

* p value for deaths during treatment.

† p value for total deaths.

Seven (4%) people died over the course of the 2-month treatment period and 2-year follow-up period among the drug-susceptible tuberculosis cohort (two [3%] patients in the B_load_PaZ group, three [5%] patients in the B_200_PaZ group, and two [3%] patients in the HRZE group). Three of these deaths occurred during treatment (one [2%] in the B_load_PaZ group on day 5, one [2%] in the B_200_PaZ group on day 23, and one [2%] in the HRZE group on day 44). For these three patients, the cause of death was reported as pneumothorax for both of the B_load_PaZ and B_200_PaZ deaths, and acute liver and renal failure for the patient receiving HRZE. None of the deaths were considered to be related to treatment. Serious treatment-related adverse events affected two (3%) patients in the B_load_PaZ group and one (2%) patient in the HRZE group.

Three (5%) of 60 patients with rifampicin-resistant tuberculosis receiving BPaMZ had a peak ALT or AST of at least 5 × ULN, and two (3%) patients withdrew because of adverse events. For both of these patients, the withdrawal was due to elevated liver enzymes. No recorded QTc intervals of more than 500 ms were recorded among patients with rifampicin-resistant tuberculosis. Four (7%) people died in the BPaMZ group: two deaths were considered to be due to tuberculosis, one was due to cor pulmonale, and one was due to drowning. All deaths occurred after treatment and none was related to treatment. Serious treatment-related adverse events affected two (3%) patients in the BPaMZ group.

More details on the incidence of treatment-emergent adverse events by treatment group (for both drug-susceptible tuberculosis and rifampicin-resistant tuberculosis), including treatment withdrawals and relatedness assessments by the site doctors, can be found in the appendix (p 16). A full listing of deaths during the trial can also be found in the appendix (p 15).

## Discussion

This study investigated the bactericidal activity and safety of three experimental pretomanid and bedaquiline-containing regimens for the treatment of pulmonary tuberculosis. Both the bedaquiline-containing treatment groups for drug-susceptible tuberculosis demonstrated superior bactericidal activity over HRZE; this effect was maintained even when a simplified dosing schedule was used for bedaquiline. However, the highest gain in bactericidal activity at day 56 of treatment compared with HRZE was seen with BPaMZ used to treat pyrazinamide-susceptible rifampicin-resistant tuberculosis. The safety signal was similar for patients taking BPaMZ and HRZE, although more events were seen for patients taking the other two experimental regimens than for those receiving HRZE.

The BPaMZ regimen used to treat rifampicin-resistant tuberculosis showed superior bactericidal activity compared with HRZE in drug-susceptible tuberculosis. This regimen constitutes a much lower pill burden than the currently recommended WHO rifampicin-resistant tuberculosis treatment options and does not involve the use of injectable drugs.

Globally, the proportion of successful outcomes from current rifampicin-resistant tuberculosis treatment is only 54%.^1^ It is well recognised that this low proportion is due to a combination of the logistical challenges in delivering therapy over a long period of time, toxic effects from drugs (especially related to the injectable drugs), and use of less efficacious drugs than HRZE. BPaMZ could help to address these shortcomings as a simplified regimen that would be less demanding to deliver than even the 9-month WHO rifampicin-resistant tuberculosis regimen, provided its long-term efficacy with a well defined toxicity monitoring schedule can be shown in phase 3 trials. There is already evidence that rifampicin-resistant tuberculosis regimens containing bedaquiline can achieve success rates of 77% and reduce disease-associated mortality.^21^, ^22^

The rapid fall in the rifampicin-resistant tuberculosis bacillary load seen in the time to culture negativity analysis in MGIT suggests a potent sterilising effect and also the potential for BPaMZ to shorten the duration of treatment for drug-susceptible tuberculosis.

Another reason to support the use of BPaMZ in the treatment of drug-susceptible tuberculosis is isoniazid monoresistance in active tuberculosis: the global prevalence is estimated to be approximately 8% of all new cases, and use of HRZE leads to worse outcomes when treating isoniazid mono-resistant strains than when treating fully susceptible strains.^23^ However, treatment outcomes involving drug-susceptible tuberculosis and those with resistant organisms need to be assessed in larger prospective trials.

The efficacy of BPaMZ was similar in HIV-positive and HIV-negative patients, but there was a reduction in bactericidal activity for patients with pyrazinamide-resistant rifampicin-resistant tuberculosis (although this decreased bactericidal activity was still greater than that of HRZE). HIV-positive patients with active tuberculosis are more likely to have treatment failure in cases of both drug-susceptible and drug-resistant tuberculosis, although this is mitigated to a large degree by antiretroviral therapy,^24^ or to have poor adherence to treatment, often due to drug toxicities.^25^, ^26^

In light of these findings, the preserved bactericidal activity with BPaMZ in HIV-positive patients is encouraging but the numbers were small and it is not possible to draw firm conclusions. Pyrazinamide resistance is also associated with worse outcomes in rifampicin-resistant tuberculosis treatment,^27^ and approximately 60% of rifampicin-resistant tuberculosis cases are thought to be pyrazinamide resistant.^28^ If this sustained bactericidal activity in patients with both HIV and pyrazinamide-resistant rifampicin-resistant tuberculosis results in improved long-term treatment outcomes, then BPaMZ could improve treatment success among these patients.

The simplified dosing schedule of 200 mg daily for bedaquiline to treat drug-susceptible tuberculosis showed similar efficacy to the B_load_PaZ regimen using the established dosing approach, and there are implications from this for other bedaquiline-containing rifampicin-resistant tuberculosis treatment regimens used elsewhere.

A daily dosing schedule could help to reduce the burden on both patients and health-care providers, and it has been shown that simplified dosing schedules are associated with improved adherence to treatment in other conditions.^29^, ^30^ The daily-dosing approach was used to treat patients with drug-susceptible tuberculosis and those with rifampicin-resistant tuberculosis in this study, performing better than HRZE against drug-susceptible tuberculosis in both the primary and secondary efficacy endpoints. Additionally, there was no indication of a safety signal with daily bedaquiline dosing and the addition of moxifloxacin was not associated with higher frequency of QT prolongation on ECG monitoring, and this finding is in keeping with other data relating to the low incidence of cardiac arrhythmias associated with bedaquiline use in the field.^31^

Over 8 weeks of treatment, the safety profile of BPaMZ was similar to that of HRZE, whereas the incidence of peak liver enzyme elevations was higher among patients with drug-susceptible tuberculosis treated with bedaquiline-containing and pretomanid-containing drugs. The proportion of HIV-positive patients, the baseline weight, and treatment adherence was similar across all the treatment groups in the study. There was also no geographical association with toxicity and cases were spread across the trial sites. Additionally, the only death from liver failure occurred in the HRZE group.

Pharmacokinetic and pharmacodynamic data relating to the trial is to be published in a separate paper, and a larger phase 3 study is required to accurately characterise the safety profile of these experimental regimens. The current therapy for treating pulmonary tuberculosis is toxic, with clinically significant hepatotoxicity in 5–30% of patients,^32^ and part of the global effort to move beyond HRZE should be motivated by the need for alternative therapies with fewer side-effects.

This study has several limitations: first, the 8-week bactericidal activity of a drug regimen is not always a reliable indicator of a regimen's ability to improve treatment outcomes or shorten treatment duration. We accept that there are limitations to the current approach in tuberculosis trials of using early bactericidal activity as a means of predicting long-term treatment outcomes and are also aware of other methodological approaches that have been proposed (including modelling approaches and adaptive trial designs). However, the 8-week study remains a broadly accepted (although imperfect) means of assessing the potential of experimental regimens as candidates for phase 3 trials.

We would ultimately argue that phase 2 studies still have a place in the development of tuberculosis treatment regimens and that culture conversion in the first 2 months of treatment is associated with outcomes in phase 3 studies but that these predictions can be imprecise. The fluoroquinolone trials were latterly acknowledged as having wide CIs around the point estimates in phase 2 studies, and the phase 3 outcomes are in the lower end of these intervals. It could be argued that the method presented in this paper still has a place in tuberculosis drug development, but previous studies have involved regimens that were insufficient and our understanding of how to interpret the results was less robust than it is now.

Additionally, this trial does not address the issue of what is an appropriate continuation phase and the continuation phase in the previous fluoroquinolone trials might have been inadequate. Furthermore, this study involves regimens that contain several experimental drugs; therefore, there is more equipoise over the regimens' ability to shorten tuberculosis treatment. Also, the overall small numbers of HIV-positive and female patients might reduce the generalisability of these findings.

Finally, caution needs to be applied when comparing the results from a randomly assigned group of patients with drug-susceptible tuberculosis to those seen among the rifampicin-resistant tuberculosis patients, who were not randomly assigned to the treatment. The rifampicin-resistant tuberculosis group of the trial was initially included as an exploratory arm to use the trial facilities as the setting to gather efficacy and safety data relating to the BPaMZ regimen. The presumed susceptibility to bedaquiline and pretomanid, with molecular testing for pyrazinamide and fluoroquinolone susceptibility, provided the scientific and ethical justification for enrolling these patients in an additional group without randomisation.

The comparisons across the treatment groups were also considered reasonable in this case because the patients recruited into the study were all enrolled on the basis of the same inclusion and exclusion criteria, with no significant differences seen in the baseline characteristics of the patients recruited, although this does not mean that there were not differences.

One issue that should be highlighted is the higher proportion of patients with rifampicin-resistant tuberculosis receiving ART at baseline than patients with drug-susceptible tuberculosis (64% *vs* 0–10%) and, as mentioned previously, ART can improve treatment outcomes among HIV-positive patients. Furthermore, if all of the treatment groups are considered to be susceptible to the regimen (noting the pyrazinamide resistance of the rifampicin-resistant tuberculosis) then the rationale was applied that comparisons were not only between similar patients but also between similar infections.

In conclusion, the BPaMZ regimen showed greater 8 week bactericidal activity against rifampicin-resistant tuberculosis than did HRZE against drug-susceptible tuberculosis in this phase 2b study. This all-oral regimen could overcome some of the shortcomings of existing rifampicin-resistant tuberculosis therapy and also possibly shorten treatment of drug-susceptible tuberculosis. The data presented here suggest that further investigation of treatment outcomes and the safety profile of BPaMZ is merited in both rifampicin-resistant tuberculosis and drug-susceptible tuberculosis, as is the further study of the efficacy and safety of the daily dosing schedule of bedaquiline in other regimens. These findings are a step forward in the development of the simple, efficacious, and safe treatment options needed in the treatment of drug-susceptible tuberculosis and rifampicin-resistant tuberculosis to meet the ambitious goals of WHO's End TB strategy.

## Data sharing

Qualified researchers may contact the corresponding author (CDT) to obtain specific de-identified clinical trial data with the permission of TB Alliance.
