# Supplementary material for: Bedaquiline, moxifloxacin, pretomanid, and pyrazinamide during the first 8 weeks of treatment of patients with drug-susceptible or drug-resistant pulmonary tuberculosis: a multicentre, open-label, partially randomised, phase 2b trial
Source: Lancet Respir Med. 2019 Dec;7(12):1048–58. doi: 10.1016/S2213-2600(19)30366-2 (PMC7641992; doi:10.1016/S2213-2600(19)30366-2)
Supplement: Supplementary appendix [file mmc1.pdf]

THE LANCET
Respiratory Medicine

## Supplementary appendix

This appendix formed part of the original submission and has been peer reviewed. We post it as supplied by the authors.

Supplement to: Tweed CD, Dawson R, Burger DA, et al. Bedaquiline, moxifloxacin, pretomanid, and pyrazinamide during the first 8 weeks of treatment of patients with drug-susceptible or drug-resistant pulmonary tuberculosis: a multicentre, open-label, partially randomised, phase 2b tria. *Lancet Respir Med* 2019; published online Nov 12. http://dx.doi.org/10.1016/S2213-2600(19)30366-2.

# NC-005 Supplementary Material


## 1. Acknowledgements

**TASK Applied Science, Cape Town:**
Andreas H Diacon (PI)
Madeleine Lourens (SI)
Florian Von Groote-Bidlingmaier (SI)

**University of Cape Town Lung Institute, Cape Town:**
Rod Dawson (National PI)
Kim Narunsky (SI)

**Clinical HIV Research Unit, Helen Joseph Hospital, Johannesburg:**
Mohammed Rassool (PI)
Noluthando Mwelase (SI)

**The Aurum Institute: Tembisa Hospital, Tembisa:**
Modulakgotla Sebe (PI)

**Klerksdorp Tshepong Hospital, Klerksdorp:**
Ebrahim Variava (PI)
Neil Matinson (SI)
Tumelo Molantoa (SI)

**Ifakara Health Institute Bagamoyo Research and Training Centre, Bagamoyo:**
Frederick Haraka (PI)
Klaus Reither (Co-PI)
Mohamed Sasamalo (SI/Mycolab Manager)

**Mbeya Medical Research Centre, Mbeya:**
Nyanda Elias (PI)
Christina Manyama (SI)
Emanual Sichone (Mycolab Scientist)

**University of Witwatersrand Clinical HIV Research Unit, Johannesburg:**
Francesca Conradie (PI)
Pauline Howell (SI)

**THINK, Durban:**
Suzanne Staples (PI)

**Uganda Case Western Reserve University Research Collaboration, Kampala:**
Alphonse Okwera (PI)
Brenda Okware (SI)

We also thank Robert Schall (University of the Free State and IQVIA) and Sean Van Der Merwe (University of the Free State) for their input in the statistical analysis of the efficacy data.

## 2. Ethics Approvals

| Center | Name/address of IEC/IRB | Name of Chairperson | Investigator |
|---|---|---|---|
| 001 | **PHARMA-Ethics Independent Research Ethics Committee**<br>123 Amcor Road<br>Lyttelton Manor 0157<br>**South Africa** | CSJ Duvenhage | Andreas H Diacon |
| 002 | **University of Cape Town**<br>**Faculty of Health Sciences**<br>**Human Research Ethics Committee**<br>Room E52-24 Old Main Building Groote Schuur Hospital Observatory 7925<br>Cape Town<br>**South Africa** | M Blockman | Rodney Dawson |
| 003 | **University of the Witwatersrand**<br>**Human Research Ethics Committee**<br>8 Blackwood Avenue<br>Park Town 2193<br>**South Africa** | PE Cleaton-Jones | Mohammed S Rassool |
| 004 | **University of the Witwatersrand**<br>**Human Research Ethics Committee**<br>8 Blackwood Avenue<br>Park Town 2193<br>**South Africa** | PE Cleaton Jones | Modulakgotla A Sebe (previously: Uthestra Chetty & Nomagugu Ndlovu) |
| 005 | **University of the Witwatersrand**<br>**Human Research Ethics Committee**<br>8 Blackwood Avenue<br>Park Town 2193<br>**South Africa** | PE Cleaton Jones | Ebrahim Variava |
| 006 | **National Institute for Medical Research (NIMR)**<br>**National Health Research Ethics Committee**<br>3 Barack Obama Drive<br>P.O. Box 9653<br>Dar es Salaam<br>**Tanzania**<br><br>**Ifakara Health Institute Institutional Review Board**<br>P.O. Box 78373<br>Dar es Salaam<br>**Tanzania** | Mwelecele N Malecela<br><br>Mwifadhi Mrisho | Frederick Haraka |
| 007 | **National Institute for Medical Research (NIMR)**<br>**National Health Research Ethics Committee**<br>3 Barack Obama Drive<br>P.O. Box 9653<br>Dar es Salaam<br>**Tanzania**<br><br>**Mbeya Medical Research Ethics Committee**<br>Mbeya Referral Hospital<br>P.O. Box 419<br>**Ministry of Health and Social Welfare**<br>P.O. Box 9083<br>Dar es Salaam<br>**Tanzania** | Mwelecele N Malecela<br><br>Donan Mmbando | Nyanda E Ntinginya |
| 008 | **University of the Witwatersrand**<br>**Human Research Ethics Committee**<br>8 Blackwood Avenue<br>Park Town 2193<br>**South Africa** | PE Cleaton Jones | Francesca M Conradie |
| 009 | **PHARMA-Ethics Independent Research Ethics Committee**<br>123 Amcor Road<br>Lyttelton Manor 0157<br>**South Africa** | CSJ Duvenhage | Suzanne Staples (Van Vuuren) |
| 010 | **Uganda National Council for Science and Technology (UNCST)**<br>Plot 6, Ntinda Kimera Road, Kampala<br>P.O. Box 6884,<br>Kampala<br>**Uganda**<br><br>**Makerere University** | Peter Ndemere<br>(Executive Secretary) | Alphonse Okwera |

| Center | Name/address of IEC/IRB | Name of Chairperson | Investigator |
|---|---|---|---|
| | **School of Biomedical Sciences** Higher Degrees Research and Ethics Committee<br>**Uganda** | Tumwine Lynnette | |

## 3. Trial Inclusion and Exclusion Criteria

### 3.1. Inclusion Criteria

Patients were eligible for randomization or assignment to treatment if they met all of the following criteria:

1. Provided written, informed consent prior to all trial-related procedures. Male or female, aged between 18 and 75 years, inclusive.
2. Body weight (in light clothing and with no shoes) between 35 and 100 kg, inclusive.
3. Tested at the trial appointed laboratory: *M. tuberculosis* positive on molecular test (eg, GeneXpert or Hain) and sputum smear-positive pulmonary TB on direct microscopy for acid-fast bacilli (at least 1+ on the International Union Against Tuberculosis and Lung Disease/WHO scale).

For inclusion in the DS-TB treatment arms (sensitive to rifampicin based on molecular sensitivity testing), patients were to be:

a. Either newly diagnosed or untreated for at least 3 years after the cure of a previous episode (patient could have given a history of cure and previous treatment); AND
b. Previous TB treatment had to be discontinued as per exclusion criterion

For inclusion in the MDR-TB treatment arm (resistant to rifampicin based on molecular sensitivity testing), patients were to be:

a. Sensitive to moxifloxacin by molecular sensitivity testing; AND
b. Either newly diagnosed or could have previously been treated for DS-TB and/or MDR-TB (≤7 days of treatment). Previous MDR-TB treatment had to be discontinued as per exclusion criterion.

4. A chest X-ray which in the opinion of the Investigator was compatible with TB.
5. Ability to produce an adequate volume of sputum as estimated from a screening coached spot sputum sample assessment (estimated 10 mL or more overnight production).
6. Be of non-childbearing potential or using effective methods of birth control, as defined below:

**Non-childbearing potential:**

a. The patient was not heterosexually active or practiced sexual abstinence; or
b. Female patient/sexual partner had a bilateral oophorectomy, bilateral tubal ligation, and/or hysterectomy or had been postmenopausal with a history of no menses for at least 12 consecutive months; or
c. Male patient/sexual partner had a vasectomy or bilateral orchidectomy at least 3 months prior to screening.

**Effective birth control methods:**

A double contraceptive method was to be used as follows:

a. Double barrier method which could have included any 2 of the following: a male condom, diaphragm, cervical cap, or female condom (male and female condoms should not be used together); or
b. Barrier method (1 of the above) combined with hormone-based contraceptives or an intrauterine device for the female patient/partner.

Patients had to be willing to continue practicing birth control methods throughout treatment and for 6 months (both male and female patients) after the last administration of study drug or discontinuation from study drug in case of premature discontinuation.

(Note: Hormone-based contraception alone may not have been reliable when taking the study drug; therefore, hormone-based contraceptives alone could not be used by female patients or female partners of male patients to prevent pregnancy).

### 3.2. Exclusion Criteria

Patients were excluded from the trial if they met any of the following criteria:

<u>Medical Criteria</u>

1. Evidence of clinically significant (as judged by the Investigator), metabolic, gastrointestinal, cardiovascular, musculoskeletal, ophthalmological, pulmonary, neurological, psychiatric or endocrine diseases, malignancy, or other abnormalities (other than the indication being studied) including malaria. A rapid test for malaria may have been carried out if indicated.
2. Karnofsky Performance Status score of <60%.
3. Poor general condition where any delay in treatment could not be tolerated as per discretion of the Investigator.
4. Clinically significant evidence of extrathoracic TB (eg, miliary TB, abdominal TB, urogenital TB, osteoarthritic TB, TB meningitis), as judged by the Investigator.
5. History of allergy or hypersensitivity to any of the trial study drugs or related substances.

6. Known or suspected current alcohol and/or drug abuse (positive urine drug screen) or history thereof within the past 2 years that was, in the opinion of the Investigator, sufficient to compromise the safety and/or cooperation of the patient.
7. For HIV infected patients:
    a. Had a CD4+ count <100 cells/µL.
    b. With an acquired immune deficiency syndrome-defining opportunistic infection or malignancies (except pulmonary TB).
    c. Was currently treated with or needed to initiate antiretroviral (ARV) therapy which was not compatible with the allowed ARV therapies and was not considered an appropriate candidate for switching to a regimen of ARVs which was allowed, as follows:
        i. Triple nucleoside reverse transcriptase inhibitor (NRTI) based regimen consisting of zidovudine, lamivudine, and abacavir.
        ii. Nevirapine based regimen consisting of nevirapine in combination with any NRTIs.
        iii. Lopinavir/ritonavir (Aluvia™) based regimen consisting of lopinavir/ritonavir (Aluvia™) in combination with any NRTIs.
        iv. Raltegravir in combination with NRTIs.
    d. Could not ensure a 2 week interval between commencing study drug and the start of the ARV therapy.
8. Had participated in other clinical trial(s) with investigational agent(s) within 8 weeks prior to trial start.
9. Significant cardiac arrhythmia that required medication.
10. Patients with the following at screening (per measurements and reading done by Central ECG):
    a. Marked prolongation of QT/QTc interval, eg, confirmed demonstration of a QTcF or QTcB interval >450 msec at screening.
    b. History of additional risk factors for Torsade de Pointes, eg, heart failure, hypokalemia, family history of long QT Syndrome.
    c. Used concomitant medications that were known to prolong the QT/QTc interval (see exclusion criterion 19 as well as list of restricted medication in Section **Error! Reference s ource not found.**).
    d. Any clinically significant, in the opinion of the Investigator, ECG abnormality.
11. Females who were pregnant, breastfeeding, or planning to conceive a child during the trial or within 6 months of cessation of treatment. Males planning to conceive a child during the trial or within 6 months of cessation of treatment.
12. Diabetes mellitus that resulted in hospitalization in the past year.
13. Evidence of lens opacity on slit lamp ophthalmologic examination as defined by a grading of >1+ on the AREDS2 grading system.
14. For males, any history of a clinically significant abnormality in the reproductive system.

Specific Treatments

15. Previously received treatment with PA-824, bedaquiline, or moxifloxacin as part of a clinical trial.
16. For the DS-TB treatment arms: treatment with any drug active against *M. tuberculosis* within the 3 years prior to Day 1 (including but not limited to isoniazid, ethambutol, amikacin, bedaquiline, clofazimine, cycloserine, fluoroquinolones, rifabutin, rifampicin, streptomycin, kanamycin, para-aminosalicylic acid, rifapentine, pyrazinamide, thioacetazone, capreomycin, thioamides, metronidazole). Exceptions included the use of fluoroquinolones and metronidazole as short-term treatment (≤2 weeks) for non-*M. tuberculosis* infections.
    Treatment was to have been discontinued at least 3 months prior to Day 1. Patients who had previously received isoniazid prophylactically may have been included in the trial as long as that treatment was discontinued at least 7 days prior to randomization.
17. Patients with MDR-TB may have been previously treated for DS-TB with first-line TB drugs (isoniazid, rifampicin, ethambutol, pyrazinamide and/or streptomycin) and/or received ≤7 days MDR-TB treatment, provided that the treatment was discontinued at least 7 days prior to randomization. It was to be confirmed that the MDR-TB treatment could be safely stopped and the screening period was long enough to allow for a washout period of 5 times the longest half-life of the drugs.
18. Any diseases or conditions in which the use of the standard TB drugs or any of their components was contraindicated, including but not limited to acute gout, allergy to any TB drug, their components, or to the study drug.
19. Use of any drug within 30 days prior to study drug administration known to prolong QTc interval (including but not limited to amiodarone, bepridil, chloroquine, chlorpromazine, cisapride, cyclobenzaprine, clarithromycin, disopyramide dofetilide, domperidone, droperidol, erythromycin, halofantrine, haloperidol, ibutilide, levomethadyl, mesoridazine, methadone, pentamidine, pimozide,

procainamide, quinidine, sotalol, sparfloxacin, thioridazine). Exceptions may have been made for patients who had received 3 days or less of 1 of these drugs or substances, if there had been a wash-out period before administration of study drug equivalent to at least 5 half-lives of that drug or substance. Patients who had taken drugs with long elimination half-lives such as amiodarone were to be discussed with the Sponsor.

20. Use of any drugs or substances within 30 days prior to study drug administration known to be strong inhibitors or inducers of cytochrome P450 (CYP) enzymes (including but not limited to quinidine, tyramine, ketoconazole, fluconazole, testosterone, quinine, gestodene, metyrapone, phenelzine, doxorubicin, troleandomycin, cyclobenzaprine, erythromycin, cocaine, furafylline, cimetidine, dextromethorphan). Exceptions may have been made for patients that have received 3 days or less of 1 of these drugs or substances, if there had been a wash-out period before administration of study drug equivalent to at least 5 half-lives of that drug or substance.
21. Any ARVs other than allowable ARVs detailed in exclusion criterion 7 above.

<u>Based on Laboratory Abnormalities:</u>

22. Patients with the following toxicities at screening as defined by the enhanced DMID Adult Toxicity Table (November 2007) (Appendix 2 of the protocol):
    a. Serum magnesium and calcium (corrected for albumin) levels outside of the laboratory's reference range.
    b. Lipase Grade 3 or greater (>2.0 × upper limit of normal [ULN]).
    c. Creatinine Grade 2 or greater (>1.5 × ULN).
    d. Hemoglobin Grade 4 (<6.5 g/dL).
    e. Platelets >Grade 2 (under 50 × $10^9$ cells/L).
    f. Serum potassium less than the lower limit of normal for the laboratory.
    g. AST Grade 3 or greater (≥3.0 × ULN) were to be excluded.
    h. ALT Grade 3 or greater (≥3.0 × ULN) were to be excluded.
    i. Alkaline phosphatase Grade 4 (>8.0 × ULN) were to be excluded, Grade 3 (≥3.0 to 8.0 × ULN) had to be discussed with and approved by the Sponsor's medical monitor.

Total bilirubin Grade 3 or greater (≥2.0 × ULN, or ≥1.50 × ULN when accompanied by any increase in other liver function test) were to be excluded, Grade 2 (≥1.50 × ULN, or ≥1.25 × ULN when accompanied by any increase in another liver function test) had to be discussed with and approved by the Sponsor's medical monitor.

### 3.3. Reasons for Screening Failures

| Screening Failure Reason | Frequency |
|---|---|
| No smear positive test or positive molecular test | 49 |
| AFB <1+ or indeterminate | 30 |
| CD4+ <100 cells/mm3 | 25 |
| QTc duration >450ms | 25 |
| Alcohol or drug abuse | 12 |
| Withdrawal of consent | 9 |
| AREDS2 Lens Opacity (>1+) | 8 |
| ALT or AST ≥3.0xULN | 6 |
| RR-TB with moxifloxacin resistance | 6 |
| Unable to produce adequate sputum | 5 |
| Poor general condition | 5 |
| Extra-thoracic TB | 5 |
| Use of a drug that induces cytochrome P450 or prolongs QT interval | 5 |
| Previous TB treatment not permitted by exclusion criteria | 5 |
| Other clinically significant ECG abnormality | 4 |
| Elevated serum magnesium and/or calcium | 4 |
| Unwilling to take birth control or pregnant | 3 |
| Haemoglobin grade 4 (<6.5g/dl) | 3 |
| Unable to take permitted ARV regimen | 2 |
| Other | 18 |

## 4. Treatment Arms

| Treatment Arm | Active | Patient Population |
|---|---|---|
| $B_{load}$PaZ | • Days 1 to 14: 4 bedaquiline 100 mg tablets **then**<br>• Days 15 to 56: 2 bedaquiline 100 mg tablets to be taken 3 times a week on specific trial days; Days 15, 17, 20, 22, 24, 27, 29, 31, 34, 36, 38, 41, 43, 45, 48, 50, 53, and 56 **plus**<br>• Days 1 to 56: 1 × PA-824 200 mg tablet **plus**<br>• Days 1 to 56: 3 × pyrazinamide 500 mg tablets | DS-TB |
| $B_{200}$PaZ | • Days 1 to 56: 2 bedaquiline 100 mg tablets **plus**<br>• Days 1 to 56: 1 PA-824 200 mg tablet **plus**<br>• Days 1 to 56: 3 pyrazinamide 500 mg tablets | DS-TB |
| HRZE | • Days 1 to 56: Dosing per weight:<br>30 to 37 kg: 2 tablets<br>38 to 54 kg: 3 tablets<br>55 to 70 kg: 4 tablets<br>71 kg and over: 5 tablets | DS-TB |
| BMPaZ | • Days 1 to 56: 2 bedaquiline 100 mg tablets **plus**<br>• Days 1 to 56: 1 moxifloxacin 400 mg tablet **plus**<br>• Days 1 to 56: 1 PA-824 200 mg tablet **plus**<br>• Days 1 to 56: 3 pyrazinamide 500 mg tablets | MDR-TB |

Abbreviations: DS: Drug-sensitive; MDR: Multi drug-resistant; B-Pa-Z: Bedaquiline, PA-824, and pyrazinamide; HRZE: Isoniazid, rifampicin, pyrazinamide, and ethambutol; B-M-Pa-Z: Bedaquiline, moxifloxacin, PA-824, and pyrazinamide; TB: Tuberculosis
HRZE combination tablets: isoniazid 75 mg plus rifampicin 150 mg plus pyrazinamide 400 mg plus ethambutol 275 mg.

## 5. Trial visit schedule

| | | | | | | | | | | | | | | | | | | | |
|---|---|---|---|---|---|---|---|---|---|---|---|---|---|---|---|---|---|---|---|
| Visit Day | -9 to -3 | -2 | -1 | 1 | 4 | 8 | 15 | 21 | 29 | 36 | 43 | 50 | 57 | 70 | 140 | M6 SFU | M12 SFU | M18 SFU | M24 SFU |
| Week or Month | Wk -1 | | | Wk 1 | | | Wk 2 | Wk 3 | Wk 4 | Wk 5 | Wk 6 | Wk 7 | Wk 8 | Wk 10 | Wk 20 | M8 | M14 | M20 | M26 |
| | **Screening** | | | **Treatment** | | | | | | | | | | **Follow Up** | | **Telephonic Survival Follow Up** | | | |

Schematic of trial design. DS: Drug-sensitive; M: Month; MDR: Multi drug-resistant; PK: Pharmacokinetic; SFU: Survival follow-up; t.i.w: 3 times a week; WK: Week.

Upon treatment completion, the patients with DS-TB were to be provided with sufficient doses of standard of care TB treatment, as appropriate, to cover the time period from attending their last visit at the trial center until their scheduled visit at the TB clinic. All patients with DS-TB and MDR-TB were to be referred to the local community TB clinic for standard anti-TB chemotherapy according to the National TB Guidelines. The patients were provided with a referral letter to take with them to the TB clinic and a follow-up call was to be made by the trial center staff to the TB clinic to determine if the patient attended the clinic on the date as arranged.

| Period | Screening | | | Treatment | | | | | | | | | | | | | Follow Up | |
|---|---|---|---|---|---|---|---|---|---|---|---|---|---|---|---|---|---|---|
| Visit Day [a] | (-9 to -3)[b] | -2[b] | -1 | 1 | 4 | 8 | 14[h] | 15 | 22 | 29 | 36 | 43 | 50 | 56[h] | 57 | Early Withdrawal[r] | 70 | 140 |
| Week | | -1 | | 1 | | | | 2 | 3 | 4 | 5 | 6 | 7 | | 8 | | 10 | 20 |
| Written Informed Consent | X | | | | | | | | | | | | | | | | | |
| Inclusion/Exclusion | X | | | X | | | | | | | | | | | | | | |
| Demography | X | | | | | | | | | | | | | | | | | |
| Medical & Treatment History | X | | | | | | | | | | | | | | | | | |
| 12-Lead ECG[c] | X | | | X | | | | X | | X | | X | | | X | X | X | |
| Laboratory Safety Tests[d] | X | | X | X | | X | | X | X | X | X | X | X | | X | X | X | |
| Urine Pregnancy Test[e] | X | | | | | | | | | X | | | | | X | X | X | X |
| HIV and CD4 Count[f] | X | | | | | | | | | | | | | | | | | |
| Urine Drug Screen[g] | X | | | | | | | | | | | | | | | | | |
| Karnofsky Score | X | | | | | | | | | | | | | | | | | |
| TB Symptom Profile (TSP) Questionnaire[S] | X | | | | | | | X | | X | | | | | X | X | | |
| Pharmacokinetic Sampling[h] | | | | X | X | X | X | X | X | X | X | X | X | X | X | X | X | |
| Chest X-ray[i] | X[i] | ( X[i]) | ( X[i]) | | | | | | | | | | | | | | | |
| Vital Signs[j] | X | | X | X | X | X | | X | X | X | X | X | X | | X | X | X | |
| Physical Examination - full[k] | X | | | X | | | | | | | | | | | X | X | | |
| Physical Examination – limited[k] | | | | | X | X | | X | X | X | X | X | X | | | | X | |
| Ophthalmology[l] Examination | X[l] | ( X[l]) | ( X[l]) | | | | | | | | | | | | | | | X[l] |
| Randomization/Treatment Assignment[m] | | | X[m] | X[m] | | | | | | | | | | | | | | |
| IMP Administration[n] | | | | X | X | X | X | X | X | X | X | X | X | X | | | | |
| Overnight Sputum Sample[o]<br>*Name of Sample (Day)* | | X<br>*-2* | X<br>*-1* | X<br>*1* | X<br>*3* | X<br>*7* | | X<br>*14* | X<br>*21* | X<br>*28* | X<br>*35* | X<br>*42* | X<br>*49* | | X<br>*56* | | | |
| Coached Spot Sputum Samples[p]<br>*Name of Sample (Day)* | X<br>*Screen* | X<br>*-2* | X<br>*-1* | X<br>*1* | X<br>*3* | X<br>*7* | | X<br>*14* | X<br>*21* | X<br>*28* | X<br>*35* | X<br>*42* | X<br>*49* | | X<br>*56* | | | |
| Concomitant Meds/ Other Treatments | X | X | X | X | X | X | X | X | X | X | X | X | X | X | X | X | X | |
| Adverse Events[q] | X | X | X | X | X | X | X | X | X | X | X | X | X | X | X | X | X | X |
| Referral to National TB Treatment Program | | | | | | | | | | | | | | | X | X | | |

### 5.1. Summary of Microbiology Assessments

| Sample | Type | Assessments | Comments |
|---|---|---|---|
| **Screening** | Coached Spot Sputum Sample | • Direct microscopy for acid-fast bacilli<br>• Molecular assay for identification of *M.* Tb and drug susceptibility (such as GeneXpert or MTBDR*plus*) to confirm the diagnosis of TB and distinguish between DS-TB and MDR-TB<br>• Molecular test for fluoroquinolone resistance (such as MTBDR*sl*) for MDR-TB Subjects to establish susceptibility to moxifloxacin | All to be performed at the Trial Appointed Laboratory. |
| **Baseline Overnight Sputum Samples named Day -2 and -1** | Overnight Sputum Sample | • Direct microscopy for acid-fast bacilli<br>• Molecular / antigen test to confirm *M. Tb*<br>• Culture: MGIT and Solid Media (quantitative for CFU)<br>• DST : SIRE, Z<br>• MIC : J, Pa, M<br>• DNA for pncA Sequencing | Z DST resistance must be repeated to confirm. |
| **Baseline Coached Spot Sputum Samples named Day -2 and -1** | Coached Spot Sputum Sample | • Culture: MGIT and Solid Media (quantitative for CFU) | For determination of logCFU and logTTP rates of change for comparison to cultures from Overnight Sputum Sample. |
| **Overnight Sputum Sample named Days 1, 3, 7, 14, 21, 28, 35, 42, 49 and 56** | Overnight Sputum Sample | • Culture: MGIT and Solid Media (quantitative for CFU)<br>• The last positive sample from withdrawn Subjects who have not converted to culture negative status OR Subjects who are still culture positive at 8 weeks OR the first positive sample after conversion to culture negative status for subjects who have 'relapsed'*<br>  o DST : SIRE, Z<br>  o MIC: J, Pa, M | |
| **Coached Spot Sputum Sample named Days 1, 3, 7, 14, 21, 28, 35, 42, 49 and 56** | Coached Spot Sputum Sample | • Culture: MGIT and Solid Media (quantitative for CFU) | For determination of logCFU and logTTP rates of change for comparison to cultures from Overnight Sputum Sample. |

## 6. Trial Endpoints

The Overnight Sputum Samples were used to determine the primary outcome of the study.

### 6.1. Primary Endpoint

The Bactericidal Activity ($BA_{TTP}$(0-56)) as determined by the rate of change in time to sputum culture positivity (TTP) over 8 weeks of treatment in the Mycobacterial Growth Indicator Tube system, represented by the model-fitted log(TTP) results as calculated by the regression of the observed log(TTP) results over time.

### 6.2. Secondary Endpoints

- The $BA_{TTP}$(0-2) and $BA_{TTP}$(14-56) as determined by the rate of change in time to sputum culture positivity (TTP) over Days 0 to 2, and Days 14 to 56 treatment, represented by the model-fitted log(TTP) as calculated by the regression of the observed log(TTP) counts over time.
- The $BA_{CFU}$(0-56), $BA_{CFU}$(0-2) and $BA_{CFU}$(14-56) as determined by the rate of change in colony forming units (CFU) over 8 weeks of treatment represented by the model-fitted log(CFU) results as calculated by the regression of the observed log(CFU) results over time.
- Time to sputum culture conversion using data from weekly cultures through 8 weeks of treatment (separately, on solid and liquid media).
- Proportion of Subjects with sputum culture conversion at 4, 6 and 8 weeks (separately, on solid and liquid media).
- The $BA_{CFU}$(0-56, 0-2 and 14-56) and $BA_{TTP}$(0-56, 0-2 and 14-56) of $B_{load}$PaZ compared to $B_{200}$PaZ from DS-TB treatment arms.
- Investigation of the methodology of sputum sampling by comparing CFU counts and TTP results, each quantified in both Coached Spot Sputum and Overnight Sputum samples; however Overnight Sputum Samples are considered the reference samples.

## 7. Additional results

### 7.1. Descriptive Statistics of log(TTP) Over Time by Treatment Arm

| Visit | Statistic | $B_{load}PaZ$ (N=57) | $B_{200}PaZ$ (N=57) | HRZE (N=59) | BMPaZ (N=38) |
|---|---|---|---|---|---|
| BASELINE | n | 57 | 56 | 57 | 38 |
| | Mean | 1.978 | 2.001 | 1.953 | 2.073 |
| | Standard deviation | 0.110 | 0.168 | 0.105 | 0.169 |
| | Minimum | 1.740 | 1.687 | 1.698 | 1.816 |
| | Median | 1.971 | 1.957 | 1.951 | 2.067 |
| | Maximum | 2.294 | 2.510 | 2.251 | 2.406 |
| DAY 4 | n | 47 | 51 | 47 | 26 |
| | Mean | 2.158 | 2.153 | 2.187 | 2.253 |
| | Standard deviation | 0.150 | 0.225 | 0.102 | 0.136 |
| | Minimum | 1.886 | 1.544 | 1.940 | 2.037 |
| | Median | 2.146 | 2.124 | 2.204 | 2.249 |
| | Maximum | 2.588 | 3.003 | 2.436 | 2.458 |
| DAY 8 | n | 46 | 45 | 49 | 33 |
| | Mean | 2.336 | 2.297 | 2.305 | 2.404 |
| | Standard deviation | 0.165 | 0.190 | 0.111 | 0.257 |
| | Minimum | 2.017 | 1.763 | 2.086 | 1.230 |
| | Median | 2.346 | 2.279 | 2.310 | 2.435 |
| | Maximum | 3.003 | 3.003 | 2.563 | 2.738 |
| DAY 15 | n | 49 | 47 | 44 | 26 |
| | Mean | 2.468 | 2.448 | 2.406 | 2.557 |
| | Standard deviation | 0.168 | 0.159 | 0.167 | 0.179 |
| | Minimum | 2.079 | 2.097 | 1.792 | 2.182 |
| | Median | 2.444 | 2.422 | 2.403 | 2.583 |
| | Maximum | 3.003 | 3.003 | 3.003 | 3.003 |

TTP: Time to positivity. log(TTP): Logarithm of TTP to the base of 10. N = Total number of patients in the efficacy analysis population. n = Number of patients with data.

| Visit | Statistic | $B_{load}$PaZ (N=57) | $B_{200}$PaZ (N=57) | HRZE (N=59) | BMPaZ (N=38) |
|---|---|---|---|---|---|
| DAY 22 | n | 46 | 44 | 50 | 31 |
| | Mean | 2.507 | 2.540 | 2.491 | 2.654 |
| | Standard deviation | 0.352 | 0.259 | 0.191 | 0.207 |
| | Minimum | 0.602 | 1.740 | 2.093 | 2.086 |
| | Median | 2.490 | 2.497 | 2.480 | 2.652 |
| | Maximum | 3.003 | 3.003 | 3.003 | 3.003 |
| DAY 29 | n | 42 | 39 | 48 | 29 |
| | Mean | 2.595 | 2.685 | 2.568 | 2.771 |
| | Standard deviation | 0.248 | 0.247 | 0.182 | 0.173 |
| | Minimum | 1.934 | 2.371 | 2.207 | 2.455 |
| | Median | 2.550 | 2.607 | 2.539 | 2.741 |
| | Maximum | 3.003 | 3.003 | 3.003 | 3.003 |
| DAY 36 | n | 43 | 44 | 44 | 27 |
| | Mean | 2.734 | 2.714 | 2.633 | 2.862 |
| | Standard deviation | 0.268 | 0.271 | 0.274 | 0.229 |
| | Minimum | 2.049 | 2.117 | 1.748 | 2.045 |
| | Median | 2.750 | 2.679 | 2.629 | 3.003 |
| | Maximum | 3.003 | 3.003 | 3.003 | 3.003 |
| DAY 43 | n | 43 | 43 | 46 | 26 |
| | Mean | 2.826 | 2.789 | 2.735 | 2.878 |
| | Standard deviation | 0.226 | 0.225 | 0.205 | 0.228 |
| | Minimum | 2.179 | 2.301 | 2.310 | 2.223 |
| | Median | 3.003 | 2.794 | 2.690 | 3.003 |
| | Maximum | 3.003 | 3.003 | 3.003 | 3.003 |

TTP: Time to positivity. log(TTP): Logarithm of TTP to the base of 10. N = Total number of patients in the efficacy analysis population. n = Number of patients with data.

| Visit | Statistic | $B_{load}PaZ$ (N=57) | $B_{200}PaZ$ (N=57) | HRZE (N=59) | BMPaZ (N=38) |
|---|---|---|---|---|---|
| DAY 50 | n | 41 | 42 | 36 | 25 |
| | Mean | 2.813 | 2.867 | 2.807 | 2.904 |
| | SD | 0.281 | 0.180 | 0.192 | 0.200 |
| | Minimum | 1.778 | 2.490 | 2.433 | 2.286 |
| | Median | 3.003 | 3.003 | 2.862 | 3.003 |
| | Maximum | 3.003 | 3.003 | 3.003 | 3.003 |
| DAY 57 | n | 38 | 45 | 42 | 26 |
| | Mean | 2.876 | 2.924 | 2.831 | 2.995 |
| | SD | 0.216 | 0.153 | 0.246 | 0.041 |
| | Minimum | 2.265 | 2.493 | 1.987 | 2.794 |
| | Median | 3.003 | 3.003 | 2.916 | 3.003 |
| | Maximum | 3.003 | 3.003 | 3.003 | 3.003 |

TTP: Time to positivity. log(TTP): Logarithm of TTP to the base of 10. N = Total number of patients in the efficacy analysis population. n = Number of patients with data.

### 7.2. Sub-group Analyses by Treatment Arm

| Subgroup | Level | Treatment Group | n | Mean Posterior Estimate | 95% BCI |
|---|---|---|---|---|---|
| HIV Status | Negative | $B_{load}$PaZ | 49 | 5.05 | 4.41;5.75 |
| | | $B_{200}$PaZ | 46 | 5.14 | 4.57;5.78 |
| | | HRZE | 49 | 4.09 | 3.71;4.49 |
| | | BMPaZ | 23 | 5.23 | 4.43;6.19 |
| | Positive | $B_{load}$PaZ | 8 | 3.94 | 2.58;5.43 |
| | | $B_{200}$PaZ | 10 | 5.90 | 3.85;8.29 |
| | | HRZE | 10 | 3.39 | 1.54;5.01 |
| | | BMPaZ | 14 | 5.21 | 4.18;6.33 |
| Cavities | Absent | $B_{load}$PaZ | 14 | 4.24 | 2.49;6.12 |
| | | $B_{200}$PaZ | 10 | 7.70 | 3.75;12.92 |
| | | HRZE | 14 | 4.18 | 2.56;5.88 |
| | | BMPaZ | 2 | --- | --- |
| | Present | $B_{load}$PaZ | 43 | 5.19 | 4.35;6.07 |
| | | $B_{200}$PaZ | 46 | 5.02 | 4.33;5.75 |
| | | HRZE | 45 | 4.00 | 3.41;4.58 |
| | | BMPaZ | 35 | 5.28 | 4.50;6.09 |
| Gender | Female | $B_{load}$PaZ | 14 | 5.72 | 4.39;7.23 |
| | | $B_{200}$PaZ | 11 | 8.80 | 6.71;11.57 |
| | | HRZE | 14 | 4.59 | 3.73;5.51 |
| | | BMPaZ | 13 | 5.48 | 4.11;7.15 |
| | Male | $B_{load}$PaZ | 43 | 4.61 | 3.99;5.29 |
| | | $B_{200}$PaZ | 45 | 4.52 | 4.06;5.01 |
| | | HRZE | 45 | 3.86 | 3.41;4.30 |
| | | BMPaZ | 24 | 5.13 | 4.41;5.95 |

Bactericidal activity characterized by joint Bayesian NLME modeling of the daily rate of change in mean $\log_{10}$TTP (efficacy analysis population), adjusted for A) HIV status B) Cavities and C) Gender and Treatment Group interactions. Treatment: E = Ethambutol, H = Isoniazid, B = Bedaquiline, M = Moxifloxacin, Pa = Pretomanid, R = Rifampicin, Z = Pyrazinamide. BCI: Bayesian credibility interval. NLME: Non-linear mixed effects. TTP: Time to positivity. log(TTP): Logarithm of TTP to the base of 10. n = Number of patients with data. Inferential statistics: Calculated from Bayesian NLME regression models fitted to log(TTP) collected from sputum samples (observed from Day 0 to Day 56) of all patients jointly.

## 7.3. Deaths in the Trial, including 24-month Survival Follow-up

| Description | Statistic | $B_{load}$PaZ (N=59) | $B_{200}$PaZ (N=60) | HRZE (N=61) | BMPaZ (N=60) | Total (N=240) |
|---|---|---|---|---|---|---|
| Deaths | n (%) | 2( 3.4) | 3( 5.0) | 2( 3.3) | 4( 6.7) | 11( 4.6) |
| Cause of death | | | | | | |
| Acute liver and renal failure/severe liver and kidney failure | n (%) | 0 | 0 | 1( 1.6) | 0 | 1( 0.4) |
| Cardiovascular failure secondary to upper gastrointestinal bleeding. | n (%) | 0 | 1( 1.7) | 0 | 0 | 1( 0.4) |
| Extensive tuberculosis | n (%) | 0 | 0 | 0 | 1( 1.7) | 1( 0.4) |
| Fatal shooting | n (%) | 0 | 1( 1.7) | 0 | 0 | 1( 0.4) |
| Possible drowning | n (%) | 0 | 0 | 0 | 1( 1.7) | 1( 0.4) |
| Severe cor-pulmonale | n (%) | 0 | 0 | 0 | 1( 1.7) | 1( 0.4) |
| Severe dyspnoea related to TB disease | n (%) | 0 | 0 | 0 | 1( 1.7) | 1( 0.4) |
| Spontaneous pneumothorax/pneumothorax | n (%) | 1( 1.7) | 1( 1.7) | 0 | 0 | 2( 0.8) |
| Unknown | n (%) | 0 | 0 | 1( 1.6) | 0 | 1( 0.4) |
| Unknown (possibly HIV disease) | n (%) | 1( 1.7) | 0 | 0 | 0 | 1( 0.4) |

Treatment: E = Ethambutol, H = Isoniazid, B = Bedaquiline, M = Moxifloxacin, Pa = Pretomanid, R = Rifampicin, Z = Pyrazinamide. MDR: Multi drug-resistant. TB: Tuberculosis. n = Number of patients in each category. N = Total number of patients randomized/assigned to study drug. % = Percentage of patients in each category relative to the total number of patients randomized/assigned to study drug.

## 7.4. Treatment-emergent Adverse Events According to Study Arm

| Description | Statistic | $B_{load}$PaZ (N=59) | $B_{200}$PaZ (N=60) | HRZE (N=61) | BMPaZ (N=60) | Total (N=240) |
|---|---|---|---|---|---|---|
| Patients with at least one TEAE | n (%) | 50 (84.7) | 45 (75.0) | 44 (72.1) | 57 (95.0) | 196 (81.7) |
| Patients with at least one drug-related TEAE | n (%) | 38 (64.4) | 29 (48.3) | 29 (47.5) | 46 (76.7) | 142 (59.2) |
| Patients with at least one TEAE leading to death | n (%) | 1 (1.7) | 1 (1.7) | 1 (1.6) | 0 | 3 (1.3) |
| Patients with at least one serious TEAE | n (%) | 4 (6.8) | 3 (5.0) | 4 (6.6) | 4 (6.7) | 15 (6.3) |
| Patients with at least one serious drug-related TEAE | n (%) | 2 (3.4) | 0 | 1 (1.6) | 2 (3.3) | 5 (2.1) |
| Patients with at least one TEAE leading to discontinuation of study drug | n (%) | 6 (10.2) | 5 (8.3) | 2 (3.3) | 2 (3.3) | 15 (6.3) |
| Patients (who completed treatment) with at least one TEAE leading to early withdrawal from study | n (%) | 0 | 0 | 0 | 0 | 0 |
| Patients with at least one Grade III TEAE | n (%) | 19 (32.2) | 17 (28.3) | 14 (23.0) | 13 (21.7) | 63 (26.3) |
| Patients with at least one Grade IV TEAE | n (%) | 8 (13.6) | 7 (11.7) | 2 (3.3) | 1 (1.7) | 18 (7.5) |
| Patients with at least one liver-related TEAE | n (%) | 6 (10.2) | 7 (11.7) | 4 (6.6) | 9 (15.0) | 26 (10.8) |
| Patients with at least one serious liver-related TEAE | n (%) | 2 (3.4) | 0 | 2 (3.3) | 2 (3.3) | 6 (2.5) |

Treatment: E = Ethambutol, H = Isoniazid, B = Bedaquiline, M = Moxifloxacin, Pa = Pretomanid, R = Rifampicin, Z = Pyrazinamide. AE: Adverse event. DMID: Division of Microbiology and Infectious Diseases. TEAE: Treatment-emergent adverse event. n = Number of patients with at least 1 TEAE in each category (patients with multiple TEAEs in each category were counted only once in each category). N = Total number of patients in the Safety analysis population. % = Percentage of patients with at least 1 TEAE in each category relative to the total number of patients in the Safety analysis population. TEAEs: Defined as AEs which started or worsened on or after the first study drug administration up to and including the Day 70 follow-up visit (or up to and including 14 days after last study drug administration for patients not having the Day 70 follow-up visit). TEAEs leading to death: Defined as TEAEs for which outcome was indicated as ‘fatal’. Serious TEAEs: Defined as TEAEs for which serious was indicated as ‘yes’. TEAEs leading to early withdrawal from study: Defined as TEAEs for which study discontinuation was indicated as ‘yes’. TEAEs leading to discontinuation of study drug: Defined as TEAEs for which action taken with study drug was indicated as ‘study drug stopped’. Drug-related TEAEs: Defined as TEAEs for which relationship to study drug was indicated as ‘possible’, ‘probable’, ‘certain’ or missing. Grade III TEAEs: Defined as TEAEs for which the severity (DMID grade) was indicated as ‘Grade 3 (severe)’. Grade IV TEAEs: Defined as TEAEs for which the severity (DMID grade) was indicated as ‘Grade 4 (potentially life-threatening)’ or missing. Liver-related AEs: Defined as any AE with a high level group term of “Hepatic and Biliary Neoplasms Benign”, “Hepatic and Hepatobiliary Disorders”, “Hepatobiliary Disorders Congenital”, “Hepatobiliary Neoplasms Malignant and Unspecified”, “Hepatobiliary Investigations” or “Hepatobiliary Therapeutic Procedures”.
